# Erratum To: pKAMA-ITACHI Vectors for Highly Efficient CRISPR/Cas9-mediated Gene Knockout in *Arabidopsis Thaliana*

**DOI:** 10.1093/pcp/pcab057

**Published:** 2021-08-23

**Authors:** Hiroki Tsutsui, Tetsuya Higashiyama

**Affiliations:** Division of Biological Science, Graduate School of Science, Nagoya University, Furo-cho, Chikusa-ku, Nagoya, Aichi 464-8602, Japan; JST ERATO Higashiyama Live-Holonics Project, Nagoya University, Furo-cho, Chikusa-ku, Nagoya, Aichi 464-8602, Japan; Division of Biological Science, Graduate School of Science, Nagoya University, Furo-cho, Chikusa-ku, Nagoya, Aichi 464-8602, Japan; JST ERATO Higashiyama Live-Holonics Project, Nagoya University, Furo-cho, Chikusa-ku, Nagoya, Aichi 464-8602, Japan; Institute of Transformative Bio-Molecules (WPI-ITbM), Nagoya University, Furo-cho, Chikusa-ku, Nagoya, Aichi 464-8601, Japan

In the originally published version of this manuscript, two errors were noted and are listed in this erratum.

The following sentences appear in the first paragraph of the introduction and should have appeared as a footnote:

The nucleotide sequences reported herein have been submitted to DDBJ under accession numbers LC189561 (p35S-Cas9), LC189562 (pX2-Cas9), LC189563 (pKIR1.0), LC189564 (pKIR1.1), LC189565 (pKI1.1) and LC189566 (pKI1.1R). Patent: a patent has been applied for on the method to drive Cas9 using the RPS5A promoter for plant genome editing (patent application number: 2016–171590).

In Figure 4F, one point of data is lacking a label. This label should read [3,1].

The Publisher apologises for these errors.

